# Review on the molecular study of the Diplozoidae: analyses of currently available genetic data, what it tells us, and where to go from here

**DOI:** 10.1186/s13071-020-04417-3

**Published:** 2020-10-30

**Authors:** Quinton Marco Dos Santos, Annemariè Avenant-Oldewage

**Affiliations:** Department of Zoology, University of Johannesburg, Auckland Park, P.O. Box 524, Johannesburg, 2006 South Africa

**Keywords:** Monogenea, *Diplozoon*, *Paradiplozoon*, *Eudiplozoon*, *Sindiplozoon*, *Inustiatus*, *Afrodiplozoon*, Molecular taxonomy, Genetic characterisation, Fish parasites

## Abstract

The use of molecular tools in the study of parasite taxonomy and systematics have become a substantial and crucial component of parasitology. Having genetic characterisation at the disposal of researchers has produced mostly useful, and arguably more objective conclusions. However, there are several groups for which limited genetic information is available and, coupled with the lack of standardised protocols, renders molecular study of these groups challenging. The Diplozoidae are fascinating and unique monogeneans parasitizing mainly freshwater cyprinid fishes in Europe, Asia and Africa. This group was studied from a molecular aspect since the turn of the century and as such, limitations and variability concerning the use of these techniques have not been clearly defined. In this review, all literature and molecular information, primarily from online databases such as GenBank, were compiled and scrupulously analysed for the Diplozoidae. This was done to review the information, detect possible pitfalls, and provide a “checkpoint” for future molecular studies of the family. Hindrances detected are the availability of sequence data for only a limited number of species, frequently limited to a single sequence per species, and the heavy reliance on one non-coding ribosomal marker (ITS2 rDNA) which is difficult to align objectively and displays massive divergences between taxa. Challenging species identification and limited understanding of diplozoid species diversity and plasticity are also likely restricting factors, all of which hamper the accurate taxonomic and phylogenetic study of this group. Thus, a more integrated taxonomic approach through the inclusion of additional markers, application of more rigorous morphological assessment, more structured barcoding techniques, alongside thorough capturing of species descriptions including genetypes, genophore vouchers and reference collections in open sources are encouraged. The pitfalls highlighted are not singular to the Diplozoidae, and the study of other groups may benefit from the points raised here as well.
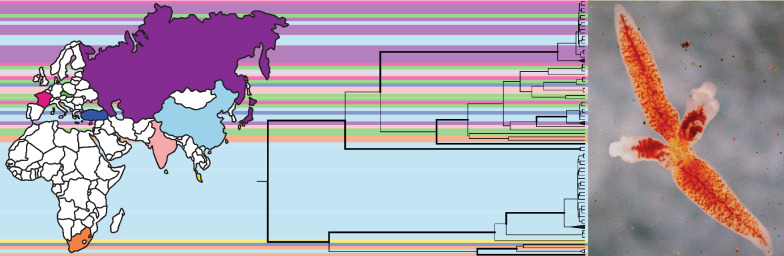

## Background

The Diplozoidae Palombi, 1949 is a fascinating and unique aquatic parasite family, with hermaphroditic monogenean pairs fusing to mature sexually, forming “X”-shaped, “Siamese” organisms, allowing for cross-fertilization [1] and supporting genetic diversity. They occur on the gills of their fish hosts, in the interhemibrachial septum of the primary lamellae, where they feed on blood and tissue, attached with specialised organs situated at their posterior ends called haptors. Within the Diplozoidae, haptors are usually disc-shaped structures with clamps, hooks, or cup-like structures, the number and shape of which is subfamily- and genus-specific. The hardened internal structures, or sclerites, of the hooks and clamps are historically considered the most crucial morphological characteristics for species identification and differentiation [2–4]. Unlike other monogeneans, species of the Diplozoidae lack sclerotised genitalia and only possess sclerites in their haptors. These sclerites are small and often difficult to accurately visualise due to their orientation and position within tissue. This, alongside the lack of other robust taxonomic features, hinder accurate identification and delimitation of species in this family. With the exception of selected species, diplozoids are considered to be host specific. They occur mostly on cyprinid fishes in freshwater systems of Eurasia and Africa, although in Africa they also parasitize characid species [5–8]. A few accounts of diplozoids infecting other fish families do exist, like Acipenseridae [3], Anguillidae [9, 10], Cichlidae [11, 12], Cottidae [3, 13], Esocidae [3], Gasterosteidae [3, 14], Gobiidae [3], Lotidae [3, 13], and Percidae [3, 9, 10, 15], even from brackish [9, 10] or marine environments [14]. Unfortunately, the majority of these records are vague, for unidentified diplozoid taxa, or have not been noted again. However, these records are intriguing and would benefit from further investigation, especially those from the Anguillidae and Percidae [3, 9, 10, 15].

Molecular approaches to the study of diplozoid parasites is a relatively new field, with members of the family only included in phylogenetic studies since 2000 [16]. Thus, only a handful of studies concerning the genetic makeup of diplozoids have been produced and 143 sequences are currently available in public data repositories and published literature (Additional file 1: Table S1). However, these sequences are representative of only 32 diplozoid taxa (as well as five unidentified species) which is less than a third of known species. The current number of known diplozoid species is uncertain as roughly 100 species have been described, of which 17 have been synonymised, 6 are considered *species inquirenda*, 20 have not been confirmed, and 55 are generally accepted, with several unidentified species mentioned in literature. Of the two subfamilies in the Diplozoidae, all genera of the Diplozoinae (*Diplozoon* von Nordmann, 1832; *Paradiplozoon* Akhmerov, 1974; *Sindiplozoon* Khotenovsky, 1981; *Inustiatus* Khotenovsky, 1978; *Eudiplozoon* Khotenovsky, 1985) have representative sequence data, constituting the majority of taxa for which data are available (31 species, 142 sequences). A single sequence for a member of the second subfamily, Neodiplozoinae, has recently been included (*Afrodiplozoon polycotyleus* (Paperna, 1973)) [17]. Thus, the only diplozoid genus for which no published molecular information is available is the monotypic *Neodiplozoon* Tripathi, 1960 (*Neodiplozoon barbi* (Tripathi, 1957)) which is the second genus within the Neodiplozoinae. Most sequence data (116 sequences) are for the second internal transcribed spacer region (ITS2) between the *5.8S* and *28S* (large) nuclear ribosomal subunits. Seven of the published ITS2 sequences include a short portion of the *28S*, while five sequences include a short portion of *18S* and the full ITS1 and *5.8S* regions, alongside the ITS2 rDNA region. The remaining 27 sequences are derived from eight gene regions: ten sequences for the universal barcoding region of the mitochondrial cytochrome *c* oxidase subunit 1 (*cox*1) gene, five sequences for the large *28S* ribosomal gene, three sequences for the small *18S* ribosomal gene, three sequences for cysteine peptidase regions (L1, L3 and B), three mitogenome sequences (from which *cox*1 can be mined) and single sequences for a cysteine peptidase inhibitor (stefin), a Kunitz-type protease inhibitor (serine), and a serine protease inhibitor (serpin) respectively.

The first molecular investigations incorporating members of the Diplozoidae [16, 18–20] were submitted prior to the proposal for a single DNA fragment for a DNA barcoding framework [21], and mostly aimed at assessing the evolutionary relationships among monogenean groups and in relation to other Platyhelminthes. The large subunit (*28S*) rRNA gene was used in most of these studies, with Jovelin & Justine [18] also amplifying the *cox*1 gene and Littlewood & Olson [19] including *18S* rRNA gene. Using *28S* rDNA, both Jovelin & Justine [18] and Olson & Littlewood [20] found diplozoid taxa to group within the polyopisthocotylean monogeneans, in the Oligochoinea (non-polystomatid polyopisthocotyleans). In both cases, the Discocotylinea, which includes the Diplozoidae, showed a close relationship to the Microcotylinea. Littlewood & Olson [19] do not refer to the position of diplozoids specifically as they only studied the relationships of the greater Polyopisthocotylea in relation to other Platyhelminthes using *18S* rDNA. Jovelin & Justine [18] noted the lack of resolution when attempting to infer evolutionary relationships between distantly related monogenean taxa using the *cox*1 gene due to the saturation of the *cox*1 alignment. Each of the above-mentioned studies produced a single sequence per marker. The sequences, five in total, represented two diplozoid species. The *cox*1, *28S* and *18S* sequences produced by Jovelin & Justine [18], Littlewood & Olson [19] and Olson & Littlewood [20] were for *Eudiplozoon kamegaii* Nishihira & Urabe, 2020 (recently separated from *Eudiplozoon nipponicum* (Goto, 1891) [22]), while the *28S* sequence produced by Mollaret et al. [16] was only identified as a *Diplozoon* sp. Additionally, the *28S* sequences deposited varied in length and coverage. Recently, sequences for several coding regions of mitochondrial DNA have been published. Jedličková et al. [23, 24] produced three sequences for endopeptidases for *E*. *kamegaii*. These enzymes (cathepsin L (L1 and L3) and B) produced phylogenies which distantly related this diplozoid to other Platyhelminthes and the authors hinted at some interesting clues about the evolution of these parasites. Sequence data for three protease (peptidase) inhibitors have also been produced for the same species (*E*. *kamegaii*). These include the cysteine peptidase inhibitor (stefin) analysed by Ilgová et al. [25], the Kunitz protease inhibitor (KT1) studied by Jedličková et al. [26], and the Serine protease inhibitor (serpin) studied by Roudnický et al. [27]. As most of the abovementioned sequence data were for a single species, little can be deduced regarding the barcoding potential of the relative regions. Zhang et al. [28] analysed the mitogenomes of three distinct diplozoids and constructed phylogenies based on all available mitogenomic data for Platyhelminthes, 21 species in total. In contrast to previous studies, diplozoid (Discocotylinea) taxa grouped sister to species of the Chauhaneidae (Gastrocotylinea) and Microcotylidae (Microcotylinea) in all cases, indicating that mitogenomic analyses might provide more insightful results for interrelationships of monogenean suborders [28].

As mentioned, most sequence data for the Diplozoidae were generated for the ITS2 fragment. The first of these sequences were published in 2001, when both Sicard et al. [29] and Matejusová et al. [4] used this marker to distinguish diplozoid species. These authors did not state why this marker was selected for their investigations, but both concluded that ITS2 rDNA can be applied to discriminate between distinct diplozoid species. Since then, almost all studies that included molecular assessment of diplozoids used only this marker, particularly studies related to taxonomy and phylogeny. The discriminatory power of ITS2 rDNA has been used to identify, describe, and re-describe several diplozoid species. It has been speculated that the identification of the parasites from which some of these ITS2 sequences were generated, as well as those for other markers, are incorrect [22, 30–32], causing confusion and controversy, and thus requiring revision. Additionally, the usefulness of other markers to study cryptic families like the Diplozoidae has been neglected and should be assessed alongside a revision of ITS2 rDNA.

Given the limited amount of data published, the taxonomic uncertainty of some published sequences, and the disparity of the selection of genetic markers to study these parasites, the time is ripe for a comprehensive revision of available molecular information on the Diplozoidae. The aim of this review was to collect, compile, analyse, and scrutinise the currently available taxonomic genetic data for the Diplozoidae. We aimed to determine if genetic studies of species of this family are on the right track, what can be gleaned from available data, how studies could be improved in the future, and what pitfalls to avoid.

## Barcoding for species identification

The molecular study of the Diplozoidae for taxonomic purposes highlights two of the most pressing pitfalls of molecular taxonomy. The first is the use of different markers, or different regions of the same gene fragment, in isolated studies and for different species, producing data which are not comparable. The neglect of the universal barcoding gene, *cox*1, to study these parasites also makes it difficult to compare this family to most other organisms for which this marker has become the benchmark. Secondly, the incorrect, incomplete, and often subjective identification of the material of the published sequence data, with little or no supporting morphological data can cause much confusion. To assess the sequences and markers available, relative sequences were aligned using MEGA 7 [33] and pairwise distances (uncorrected p*-*distance) calculated (1000 bootstrap replicated). All sequence data for protein-coding markers are for the same species, *E*. *kamegaii*, and thus the taxonomic usefulness of these markers could not be assessed.

### 18S rDNA

Only three sequences containing substantial fragments of *18S* rDNA are available for diplozoids. The alignment of these sequences consisted of 1962 bp, with 1773 conserved, 112 variable, and no parsimony informative sites, with uncorrected p-distances between 0–7.73% (Table 1). Two of these sequences are identical and are for *E*. *kamegaii*, AJ287510 by Littlewood & Olson [19] and MF579987 by E. Turgay (unpublished). The similarity of these two sequences, one collected from the Czech Republic [19] and the other from Turkey (according to an unpublished conference abstract by Kırcalar et al. [34]), suggest that this marker might be a suitable candidate for barcoding. They are both also markedly distinct from the sequence for *Paradiplozoon hemiculteri* (Ling, 1973) (KY640614; 5.94–7.73%), which is only available from GenBank and not published otherwise. The sequences available cover similar regions of *18S* rDNA, but are markedly different in length as MF579987 (750 bp) corresponds to just more than a third of AJ287510 (1960 bp) and KY640614 (1887 bp). Five other sequences (KY290757–KY290761) contain a tiny fragment of *18S* rDNA (37 bp), but they do not overlap meaningfully with the aforementioned three sequences. These five sequences also include the full ITS1 and *5.8S* rDNA regions, but they are the only sequences to do so and are all for the same species (*P*. *hemiculteri*), thus not allowing these two regions to be assessed beyond the 0–0.59% intraspecific distances calculated.

**Table 1:** Sequence divergence (average uncorrected p-distance in %) for diplozoid taxa based on partial *18S* rDNA

GenBank ID	Species		1	2
AJ287510	*Eudiplozoon* *kamegaii*	1	–	
MF579987	*Eudiplozoon* *kamegaii*	2	0	–
KY640614	*Paradiplozoon* *hemiculteri*	3	5.94	7.73

### 28S rDNA

The five available sequences for *28S* rDNA of diplozoids contains either two or three “marker fragments” because different domains of this ribosomal region have been sequenced by different authors and are not always comparable. Sequences by Sicard et al. [29], Ahmad et al. [35, 36], and an unpublished sequence by Sofi & Ahmad (GenBank: MF460994) include a short section of the 3'-end of *28S* (C1 and D1 domains) alongside the ITS2 fragments, about 310 bp (190 bp for MF460994). These fragments do not fully overlap with any of the exclusively *28S* rDNA sequences, with the closest being that by Mollaret et al. [16] of which 76.64% (269 out of 351 bp) is covered. However, similar to the sequence by Mollaret et al. [16], they do not overlap with the fragment of Jovelin & Justine [18] at all. Nevertheless, they were included in analyses, bringing the total sequences analysed to 12. The produced alignment consisted of 1276 bp, with 728 conserved, 254 variable, and 123 parsimony informative sites, with uncorrected p-distances between 0–35.09% (Table 2). Due to the inconsistent *28S* coverage of sequences from different species and authors, erratic sequence length (351–1133 bp), and the limited number of species for which *28S* data are available, it is challenging to determine whether this marker would show enough resolution to study diplozoid diversity.

**Table 2:** Sequence divergence (average uncorrected p-distance in %) for diplozoid taxa based on partial *28S* rDNA

	GenBank ID	Species	1	2	3	4	5	6	7	8	9	10	11
1	AF311703	*Eudiplozoon kamegaii*	–										
2	AF382037	*Eudiplozoon kamegaii*	0	–									
3	AF369758	*Eudiplozoon kamegaii*	na	0	–								
4	AF369761	*Paradiplozoon bliccae*	na	5.02	4.58	–							
6	AF369760	*Paradiplozoon homoion*	na	5.34	4.87	1.31	–						
5	AF131717	*Diplozoon* sp.^a^	na	4.96	6.42	1.15	1.89	–					
7	AF369759	*Diplozoon paradoxum*	na	6.08	5.5	0.98	1.62	0	–				
8	AF973617	*Paradiplozoon aegyptense*	na	11.79	10.36	6.54	7.12	6.39	5.48	–			
9	AF973616	*Paradiplozoon kashmirense*	na	14.58	14.14	9.95	10.99	10.14	9.95	13.09	–		
10	MF460994	*Paradiplozoon kashmirense*	na	14.58	14.14	9.95	10.99	10.14	9.95	13.09	0	–	
11	MN545903	*Paradiplozoon hemiculteri*	23.61	18.09	16.67	17.24	17.42	13.08	17.74	23.02	26.35	26.35	–
12	KU519493	*Paradiplozoon* sp.	22.24	18.59	19.65	21.18	21.39	15.35	21.84	27.59	35.09	35.09	12.38

^a^Following the findings presented here, this species may represent *Diplozoon paradoxum* Nordmann, 1832

*Abbreviation*: na, sequences that do not overlap

The three sequences for *E*. *kamegaii* are identical for the regions where they do overlap, and as they were produced by different authors and collected from different sites and systems, show that this marker has barcoding potential due to its apparent intraspecific stability over vast geographical reaches. Following this, even though sequences AF131717 and AF369759 only overlap for 269 bp, they are identical and may indicate that they are of the same taxon. This means that we may be able to designate the identity of AF131717, which is only noted as *Diplozoon* sp. by its authors [16], as *Diplozoon paradoxum* Nordmann, 1832, especially when also taking into account that the specimens were collected from the type-host of *D*. *paradoxum* (captured in Additional file 1: Table S1). If this assumption is made, we can see that the intraspecific range of the combination of *28S* rDNA fragments used is 0, with the lowest interspecific distance of 0.98 observed between *D*. *paradoxum* and *P*. *bliccae*, which have been noted to be closely related but distinct taxa in previous studies [4, 29, 37]. This further supports the possible use of fragments of *28S* rDNA for barcoding of diplozoid species. Based on this notion, sequence KU519493 likely represents a diplozoid taxon not yet previously genetically characterised using *28S* rDNA, even possibly a new diplozoid taxon as no diplozoid has been recorded from *Barilius barila* (Hamilton, 1822) or *Barilius bendelisis* (Hamilton, 1807).

### cox1 mtDNA

Although ten sequences are available for *cox*1 gene fragments of diplozoids, a further three sequences can be mined from the mitogenomes produced by Zhang et al. [28] to bring the total to thirteen. The produced alignment consisted of 456 bp, with 274 conserved, 182 variable, and 133 parsimony informative sites, displaying uncorrected pairwise distances between 0.24–25% (Table 3). Sequences are between 362 and 441 bp long, i.e. approximately a third of a *cox*1 gene [38], and far less than the 658 bp fragment generally used for barcoding [21]. Most *cox*1 data available for diplozoids are for *Eudiplozoon* (7 of 13 sequences). Unfortunately, within *Eudiplozoon cox*1 data there does not seem to be a clear separation of taxa. Looking at the p*-*distances calculated in Table 3, sequences LC517176 and LC517177 are only separated by 0.51% and were both identified as *E*. *nipponicum* collected from *Carassius auratus* (Linnaeus, 1758) (*Carassius buergeri grandoculis* Temminck & Schlegel, or nigorobuna, according to Nishihira & Urabe [22]). This likely indicates that these sequences represent the same taxon. However, both these sequences are more closely related to AY009163 (2.24%) for *E*. *kamegaii* from *Cyprinus carpio* Linnaeus, 1758 than to the other sequence for *E*. *nipponicum* LC517173 from *C*. *auratus* (13.38–13.82%). Additionally, sequences LC517174 and LC517175 are separated by 3.21% even though they were both identified as *E*. *kamegaii* and collected from *C*. *carpio*, with LC517174 only separated by 0.24% from LC517173 (*E*. *nipponicum* from *C*. *auratus*). Thus, within these six *Eudiplozoon cox*1 sequences, two groupings are present, LC517176, LC517177 and AY009163, separated by 0.51–2.24%, and LC517173-LC517175 separated by 0.24–3.65%. These two groupings are separated by 13.01–14.25%. This means that there are either two species present, with up to 3.65% intraspecific distances possible in the genus, or four species, with up to 0.51% intraspecific distances possible. The final *Eudiplozoon cox*1 sequence, MG458326, is for an unidentified species from *C*. *auratus* (possibly a different host than for LC517173, LC517176-LC517177 [22]), but is 9.36–15.78% from other *Eudiplozoon* data and may represent a third (or fifth) undescribed, cryptic *Eudiplozoon* taxon. Due to the uncertainty about the species identity of some of the *Eudiplozoon cox*1 data, calculating intra- or interspecific distances are difficult, but an intrageneric distance of 0.24–15.78% was observed. All other *cox*1 diplozoid sequence data are separated by more than 17%, except for *Paradiplozoon homoion* (Bychowsky & Nagibina, 1959) (KP399595) and *Paradiplozoon gracile* (Reichenbach-Klinke, 1961) (KP399596). Their low distance (0.76%) may indicate that these species are not be entirely distinct, forming part of the ‘*Paradiplozoon homoion*-complex’, which will be discussed further on. As such, from the current *cox*1 mtDNA available for diplozoids, this marker does not seem to be ideal for species identification. Analyses of diplozoid mitogenomes by Zhang et al. [28] allowed the authors to suggest that mitogenomes might provide better resolution for phylogenetic relationships, with some faster evolving mitochondrial genes being better suited to infer evolutionary histories and population genetics compared to the more slowly evolving *cox*1 [28]. These authors were cautious in this regard, again reinforcing the need for more data.

**Table 3:** Sequence divergence (average uncorrected p-distance in %) for diplozoid taxa based on partial *cox*1 mtDNA

	GenBank ID	Species	1	2	3	4	5	6	7	8	9	10	11	12
1	LC517176	*Eudiplozoon nipponicum*	–											
2	LC517177	*Eudiplozoon nipponicum*	0.51	–										
3	AY009163	*Eudiplozoon kamegaii*	2.24	2.24	–									
4	LC517173	*Eudiplozoon nipponicum*	13.82	13.38	13.41	–								
5	LC517174	*Eudiplozoon kamegaii*	14.25	13.67	13.45	0.24	–							
6	LC517175	*Eudiplozoon kamegaii*	14.25	13.01	13.17	3.65	3.21	–						
7	MG458328	*Eudiplozoon* sp.	15.78	15.19	15.69	10.95	11.16	9.36	–					
8	MG458326	*Sindiplozoon* sp.	19.59	18.82	19.05	18	18.45	17.81	17.76	–				
9	HF565162	*Paradiplozoon* sp.^a^	20.94	20.62	20.17	20.39	19.59	20	20.6	18.36	–			
10	MG458327	*Paradiplozoon opsariichthydis*	21.63	19.73	20.45	23.11	22.78	22.6	22.37	22.59	20.35	–		
11	KP399595	*Paradiplozoon homoion*	21.34	21.46	22.41	23.94	23.74	23.74	23.99	21.72	23.28	25	–	
12	KP399596	*Paradiplozoon gracile*	21.59	21.72	22.41	23.94	23.74	23.74	23.99	21.72	23.28	25	0.76	–
13	KP340976	*Diplozoon paradoxum*	22.88	23.23	22.97	21.01	21.21	22.98	23.23	22.73	20.63	24.49	22.22	22.47

^a^This sequence represents *Paradiplozoon ichthyoxanthon* Avenant-Oldewage 2014 in Avenant-Oldewage et al. [41] and should be noted as such from here on out.

Other than MG458326 (*Eudiplozoon* sp.), there are two sequences for which full identifications are not noted and for which some additional information may be deduced. MG458326 was noted as *Sindiplozoon* sp. and collected from *Spinibarbus hollandi* Oshima, 1919, *Parabramis pekinensis* (Basilewsky, 1855) and *Mylopharyngodon piceus* (Richardson, 1846). Using the hosts, it is most probable that the species is *Sindiplozoon diplodiscus* (Nagibina, 1965) as this species has been recorded from *M*. *piceus* [3, 39]. However, it could also be *Paradiplozoon parabramisi* (Ling, 1973) which has been recorded from *P*. *pekinensis* [3, 40], although this would be unlikely due to the large p-distance between MG458326 and MG458327 (*Paradiplozoon opsariichthydis* (Jiang et al., 1984)), with both *P*. *parabramisi* and *P*. *opsariichthydis* forming part of the ‘*Paradiplozoon hemiculteri*-complex’ based on ITS2 rDNA, which will be discussed further on. To our knowledge, no diplozoid has been recorded and fully identified from *S*. *hollandi*, and thus this may either be a new host for either of the aforementioned diplozoid species, or indicate that the specimens in question represent a new species. The other sequence not fully identified is HF565162 for *Paradiplozoon* sp. This sequence was produced by the present authors during the description of *Paradiplozoon ichthyoxanthon* Avenant-Oldewage, 2014 in Avenant-Oldewage et al. [41] and prematurely uploaded. This sequence is thus representative of *P*. *ichthyoxanthon* and can be considered the *cox*1 mtDNA paragenetype for the species. The present publication will be used to update its GenBank record to avoid future confusion (captured in Table 3 and Additional file 1: Table S1).

### ITS2 rDNA

Diplozoid sequences containing ITS2 rDNA fragments range from 577 bp (KT781100 (unpublished and unidentified)) to 1762 bp (KY290757–KY290761 (*P*. *hemiculteri*, unpublished)), depending on the primers used. All available ITS2 sequence data were compiled, primers removed where required, *5.8S* and *28S* ends trimmed (based on binding regions of primers D and B1 [42]), and aligned using MAFFT [43] (*via* EBI) to remove subjectivity. The produced alignment contained 116 sequences and consisted of 814 bp, with 225 conserved, 569 variable, and 469 parsimony informative sites. Distances of up to 39.72% can be seen between diplozoid taxa, while many sequences for distinct taxa are identical or have very little variation (Additional file 2: Table S2). In Table 4, the pairwise distances between the six diplozoid genera (intergeneric) for which ITS2 data are available are given alongside their intrageneric distances. The intrageneric distance of *Inustiatus* is very low (0.42%), but the two taxa designated by Gao et al. [40], *Inustiatus inustiatus* (Nagibina, 1965) and *Inustiatus aristichthysi* (Ling, 1973), were synonymised by Khotenovsky [3] in 1985, effectively producing a monotypic genus. Thus, the intrageneric distance of *Inustiatus* represents the intraspecific distance for *I*. *inustiatus* as well. This means that ITS2 sequence data for *Sindiplozoon*, *Afrodiplozoon* and *Inustiatus* are each only represented by a single taxon. *Eudiplozoon* also has a low intrageneric distance, but this distance represents two taxa, *E*. *nipponicum* and *E*. *kamegaii*, with intraspecific distances of up to 0.96% and an interspecific distance of 3.09–3.9%, supporting the distinctness of these two species. Sequence data for these genera (*Inustiatus*, *Eudiplozoon*, *Sindiplozoon* and *Afrodiplozoon*) have plausible intraspecific distances (< 1%), indicating that all five taxa involved are likely distinct. Additionally, the intrageneric range of *Eudiplozoon* is far lower than the lower limits of the intergeneric distances for these four genera (> 20%), possibly confirming the genetic validity of these genera. On the other hand, the low intergeneric distance between *Diplozoon* and *Paradiplozoon* may indicate the opposite. Additionally, the high upper limit of the intrageneric distance for *Paradiplozoon* is concerning as this is higher than the lower intergeneric limits between all genera.

**Table 4:** Inter- and intrageneric sequence divergence (average uncorrected p-distance in %) based on ITS2 rDNA

	*Diplozoon*	*Paradiplozoon*	*Sindiplozoon*	*Eudiplozoon*	*Inustiatus*	*Afrodiplozoon*
*Diplozoon*	**0–15.42**					
*Paradiplozoon*	4.34–39.72	**0–39.61**				
*Sindiplozoon*	15.47–22.83	15.44–36.3	^**a**^			
*Eudiplozoon*	19.26–27.87	18.76–37.57	20.52–24.47	**0–3.9**		
*Inustiatus*	25.25–32.05	25.04–38.86	25.57	22.49–27.11	**0.42**	
*Afrodiplozoon*	26.6–32.38	13.49–32.55	29.29	30.39–32.17	32.83	^**a**^

^a^No intrageneric distance possible as only a single sequence is available for genus.

*Note*: Intrageneric distance is indicated in bold

Within *Paradiplozoon*, intraspecific distances of 0–3.05% can be seen, while interspecific distance of as low as 0% are also seen (Additional file 2: Table S2). This suggests that there is something amiss with either the available data, the identification of specimens, or the viability of ITS2 rDNA to differentiate diplozoid species, and thus needs to be reassessed. There are two groupings of species for which interspecific distances of 0 were calculated, the ‘*P. homoion*-complex’ which may consist of three species occurring across Eurasia, and the ‘*P. hemiculteri*-complex’ which may consist of six species and occur in China. These two species complexes showed interspecific distances of 0–1.32% and 0–3.05% respectively and will be discussed in full further on. When these species-complexes are not considered, the intraspecific distances observed for *Paradiplozoon* become 0–0.44%, while interspecific distances of 1.9–39.72% are calculated for the genus. These ranges do not overlap and as such a robust value of 1.5% can be used as the limit at which different diplozoid species can be differentiated using ITS2 rDNA. The upper intraspecific limit also compliments that seen in other diplozoid genera (< 1%), supporting this observation. If these limits are used to try and designate sequences which were not fully identified, sequences MN892630–MN892639 (unpublished) are clearly distinct from other diplozoid taxa (13.79–33.04%, Additional file 2: Table S2) and thus represent a species not genetically typed using ITS2 before. Due to the lack of information in the GenBank records by Kadirden (Arken) and Cheng (Yue) for these sequences, and the fact that they are unpublished, we cannot infer much about their identity. Thus, the assumption of previous studies, that ITS2 rDNA may be a good marker for barcoding and delimitation of diplozoid species, may be correct and should continue, but only if closely related species complexes are disregarded or ideally resolved. However, the study of the evolutionary history of the Diplozoidae using this marker while species complexes are not yet fully resolved is not ideal.

## Phylogenetic studies

**Fig. 1:**
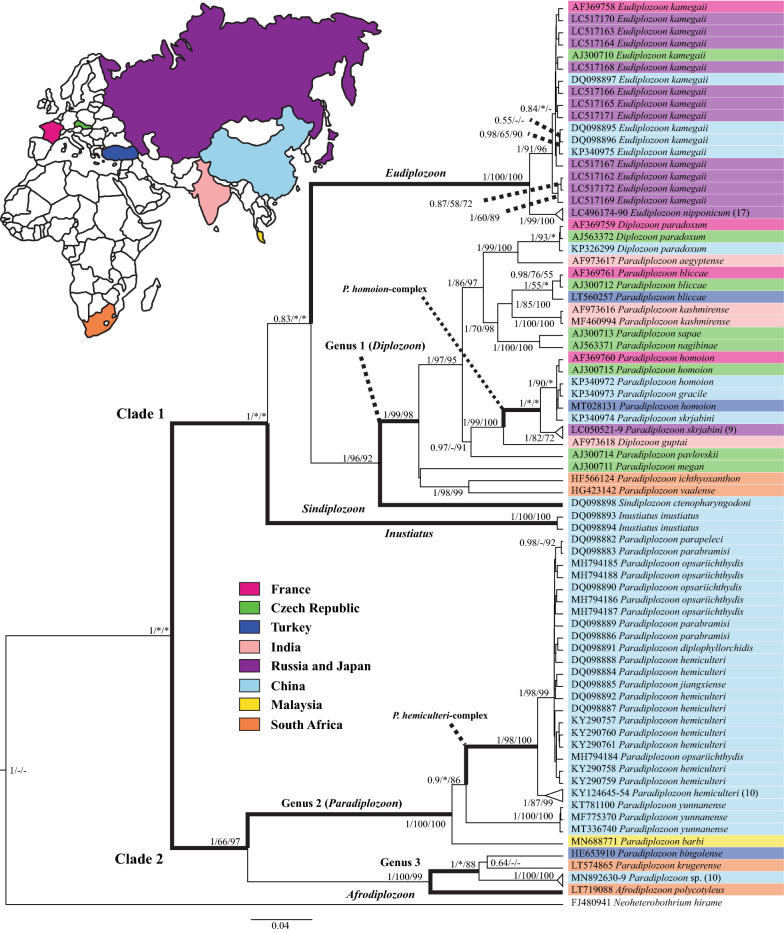
Bayesian inference analysis tree based on all available ITS2 rDNA sequences for the Diplozoidae, with *Neoheterobothrium hirame* Ogawa, 1999 used as the outgroup. Support for BI, ML, and P is indicated at nodes (BI/ML/P), with geographical origin shown in relation to colour coded map and country key. Nodes with less than 50% support for all methods are not annotated, with those that have less than 50% support in certain analyses are indicated with “**-**”. Nodes not present in either ML or P topologies are indicated with “*”. Branches with thicker formats indicate specific taxonomic groups indicating major clades, species complexes, and current or proposed genera

To produce more accurate phylogenetic analyses of the ITS2 rDNA fragment, an outgroup was added to the alignment of diplozoid taxa, *Neoheterobothrium hirame* Ogawa, 1999 (FJ480941), as selected using BLASTn [44]. Analyses of the ITS2 alignment to produce an updated evolutionary history of the Diplozoidae was done using MEGA 7 and the BEAST v2.5.0 [45] software package. In MEGA 7, maximum likelihood (ML) and parsimony (P) methods were employed to reconstruct the evolutionary history. For ML, the general time reversible (GTR) model [46] with gamma distribution (+G, parameter = 2. 2524) was selected using the Model Selection Tool in MEGA 7. The robustness of both ML and MP analyses were tested with 1000 bootstrap replicates. Using BEAST v2.5.0, Bayesian inference (BI) analyses were performed, using 10 million Markov chain Monte Carlo (MCMC) generations. Using all generated topologies, a combined and updated topology based on BI analyses of the family was produced (Fig. 1) with MCMC and bootstrap support indicated at the respective nodes (BI/ML/P). Nodes with less than 50% support for all methods are not annotated, with those that have less than 50% support in certain analyses indicated with “–”. Nodes not present in either ML or P topologies are indicated with “*”.

Jirsová et al. [32] and Fan et al. [31] produced the most recent and complete phylogeny of diplozoid parasites, which do not differ markedly when compared to the revised topology presented here (Fig. 1). Looking at the produced topology from the most basal taxa, it would appear that all diplozoid taxa are spilt into two major clades, indicated on Fig. 1 as Clade 1 and Clade 2. However, this grouping was only well supported by BI analyses. In both ML and P analyses *Inustiatus* and *Eudiplozoon* are consecutively basal to all other diplozoids, but the nodes for this were not well supported and mostly collapse at 50% consensus. The distant relation of *E*. *nipponicum* (or *E*. *kamegaii* depending on the host [22]) in both larval development [47] and molecular data to other diplozoids, prompted its use as an outgroup in several phylogenetic studies of the family [4, 29, 35, 36, 41, 48–50]. Whereas, Gao et al. [40] designated *N*. *hirame* of the Diclidophoridae as an outgroup in their analyses to produce a more accurate phylogeny, as was done presently. This concept was also utilised by Civáňová et al. [30] and Dos Santos & Avenant-Oldewage [51] in designating *Zeuxapta seriolae* (Meserve, 1938) from the related Axinidae as an outgroup. Based on the topology presented here, even though BI analyses place *Inustiatus* and *Eudiplozoon* in Clade 1, the contradiction of this by ML and P methods mean that the placement of these genera are not resolved as of yet and their use as outgroups should be avoided. However, the grouping of *Sindiplozoon* with *Paradiplozoon* and *Diplozoon* taxa in Clade 1 is consistent across all methods used, with fairly high statistical support. Most nodes within the rest of this clade are also well supported, except for nodes related to the same taxa or those in species-complexes. Clade 1 contains roughly half of the *Paradiplozoon* and all the *Diplozoon* taxa for which sequence data are available.

The second major clade of diplozoid species, or Clade 2, consists of the single sequence for *Afrodiplozoon* and the other half of *Paradiplozoon* taxa. Interestingly, the present authors speculated that *Afrodiplozoon* may have proved an ideal outgroup to the Diplozoinae as it is currently in the subfamily Neodiplozoinae, but that does not seem to be the case based on ITS2 rDNA. *Afrodiplozoon* groups sister to *Paradiplozoon* spp. from Africa and Turkey, all of which form a sister clade to Asian (Chinese and Malaysian) *Paradiplozoon* spp., placing *Afrodiplozoon* in the ingroup of Clade 2. The statistical support for the entire Clade 2 is fairly high and consistent across the three phylogenetic methods used, with the exception of nodes related to the same taxa or those in species complexes.

**Fig. 2:**
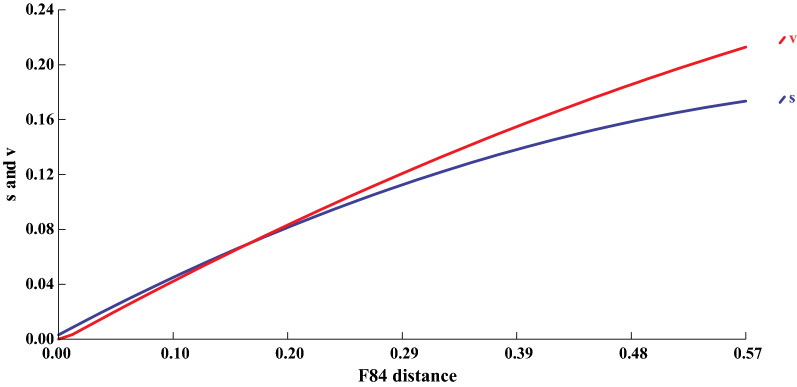
Saturation plot of the number of transitions (s, blue) and transversions (v, red) plotted against sequence divergence values (F84 distance) of the alignment of all available ITS2 rDNA sequences for the Diplozoidae

Looking at the discriminatory power of the ITS2 fragment based on the produced topology (Fig. 1), it would appear that the separation of most distinct taxa is well supported. The exceptions are the three *Paradiplozoon* taxa in Clade 1 which form part of the ‘*P. homoion*-complex’, and the six *Paradiplozoon* taxa in Clade 2 which represent the ‘*P. hemiculteri*-complex’, both of which will be discussed further on. To further assess the phylogenetic virility of the ITS2 marker, saturation of the aligned sequences was assessed using DAMBE 6.4.76 [52, 53], where the number of transitions and transversions were plotted against sequence divergence values (Fig. 2). From this plot, it is clear that the transitions of the ITS2 alignment became saturated. This may indicate that this marker is not ideal for inferring phylogenies, similar to what was seen for *cox*1 by Jovelin & Justine [18]. Thus, the suitability of other markers to study cryptic groups like the Diplozoidae need to be more vigorously investigated. Unfortunately, due to the limited number of sequences available for the other markers discussed here, topologies and saturation plots of their alignments did not show any informative results and thus much more sequence data are needed for all these markers before meaningful comparisons can be made to the suitability of ITS2 rDNA for diplozoid taxonomy.

## Genus designation based on genetic analyses

Based on the topology generated using ITS2 rDNA (Fig. 1), a few possibilities are evident in terms of the designation of genera in the Diplozoidae. First, that the entire group is monophyletic and that the Diplozoidae are monogenetic with only a single genus (*Diplozoon*). This is unlikely due to the vast genetic distances (Table 4, Additional file 2: Table S2) and morphological differences [3, 39] between current genera. Additionally, this would mean that the subfamilies Diplozoinae and Neodiplozoinae are invalid, although this is supported by the placement of *Afrodiplozoon* in Clade 2. Secondly, that species designated to *Paradiplozoon* represents two, three, or more genera. The second option is well supported by the topology and genetic distances produced here. If this were the case, one could effectively consider the grouping of *Paradiplozoon* and *Diplozoon* species in Clade 1 as Genus 1 (which would revert to the type genus *Diplozoon*), and the *Paradiplozoon* species in Clade 2 as Genus 2 (which would remain *Paradiplozoon*). The consistent grouping of *D. paradoxum* within certain *Paradiplozoon* taxa, separate from other *Paradiplozoon* taxa (Clade 2), has lead authors to speculate the paraphyly of *Paradiplozoon*, highlighting the need for a taxonomic revision of the group [30, 48, 49]. This, alongside the updated topology and the grouping of *Diplozoon guptai* Fayaz & Chishti, 2000 with *Paradiplozoon* species in Clade 1, but not sister to *D*. *paradoxum*, may support the notion that *Paradiplozoon* and *Diplozoon* in Clade 1 are monophyletic. This idea, and the subsequent notion that *Diplozoon* and *Paradiplozoon* species should all be considered *Diplozoon*, was already proposed by Sicard et al. [48], but was never applied to later studies by other authors. However, we now know that the general synonymising of these two genera was premature as there are a substantial number of *Paradiplozoon* species in Clade 2 which are clearly not monophyletic to the species included in *Diplozoon* by Sicard et al. [48].

Due to the grouping of *Afrodiplozoon* within the ingroup of Clade 2, the designation of a third genus might be possible, with Genus 2 sister to a grouping of *Afrodiplozoon* and Genus 3. This implied change in the taxonomic designation of groups will also be problematic when considering the current taxonomy of the Diplozoidae. There is currently no robust morphological criteria on which to distinguish the members of Genus 1 (*Diplozoon*), 2 (*Paradiplozoon*), and 3 confidently. And again, as A*frodiplozoon* (Neodiplozoinae) is in the ingroup of Clade 2 (Diplozoinae), the designation of subfamilies will also need to change or fall away. It would appear that according to ITS2 rDNA, the division of diplozoid genera into two subfamilies based on the number of clamp pairs [3, 39] might be entirely incorrect. If we were to use these new designations of genera and apply them to the genetic distances calculated for available sequence data, the inter- and intrageneric distances as shown in Table 5 are produced. From the distances captured in Table 5, it is clear that the limits between the newly proposed designation of genera are more separate, but still overlap slightly. The high intrageneric distance of Genus 1 (*Diplozoon*) and low intergeneric distance between Genus 2 (*Paradiplozoon*) and *Afrodiplozoon* show that no concrete limits for the designation of genera can be seen at this time using ITS2 rDNA. However, it should be made abundantly clear that these are purely speculative designations (summarised in Additional file 2: Table S3) based only on the topology and distances of ITS2 rDNA sequences presented here. This needs to be intently studied using other markers and rigorous morphological analyses, also possibly with co-phylogenetic studies alongside host material, of all genera before being considering viable. This could prove a very interesting endeavour and may re-shape the way we think about the taxonomy of cryptic groups such as the Diplozoidae.

**Table 5:** Inter- and intrageneric sequence divergence (average uncorrected p-distance in %) based on ITS2 rDNA following suggested genus designation

	Genus 1 (*Diplozoon*)	Genus 2 (*Paradiplozoon*)	Genus 3	*Sindiplozoon*	*Eudiplozoon*	*Inustiatus*	*Afrodiplozoon*
Genus 1 (*Diplozoon*)	**0–25.08**						
Genus 2 (*Paradiplozoon*)	29.46–39.72	**0–14.61**					
Genus 3	24.93–35.18	25.77–32.69	**0–9.67**				
*Sindiplozoon*	15.44–22.88	30.83–36.3	26.29–27.17	^**a**^			
*Eudiplozoon*	18.76–28.19	30.04–37.57	27.69–33.55	20.52–24.47	**0–3.9**		
*Inustiatus*	25.04–32.05	32.66–38.86	30.03–31.13	25.57	22.49–27.11	**0.42**	
*Afrodiplozoon*	25.97–32.38	29.69–32.55	13.49–15.44	29.29	30.39–32.17	32.83	^**a**^

^a^No intrageneric distance possible as only a single sequence is available for genus.

*Note*: Intrageneric distance is indicated in bold

## Phylogeography

In an attempt to determine if any phylogeographic patterns can be seen from the topology produced using ITS2 rDNA, the geographical origin of the specimens used to produce respective sequence data have been indicated on the topology of Fig. 1. As can be seen from the topology (and Additional file 1: Table S1), two genera (*Inustiatus* and *Sindiplozoon*) occur solely in Asia (China), while another occurs only in Africa (*Afrodiplozoon*). The remaining genera occur across Eurasia, with *Paradiplozoon* also occurring in Africa. Specimens of *E*. *kamegaii* included in molecular studies were collected from Turkey, France, the Czech Republic, Japan, and China, but were all collected from *C*. *carpio*. These cyprinids are indigenous to Asia, presumably introduced to Europe [54], allowing *E*. *kamegaii* to spread alongside its host. Similarly, *D*. *paradoxum* was collected and sequenced from France, the Czech Republic, Sweden (based on the above analysis of *28S* rDNA sequence AF131717), and China from the type-host *Abramis brama* (Linnaeus, 1758). This linkage between the distribution of diplozoid parasites and their type-hosts is likely due to the direct life-cycle of monogenean parasites, enabling anthropogenic distribution.

*Paradiplozoon* species on the other hand appear to have a wide distribution across Eurasia and Africa. However, some observations can be made regarding the phylogeographic properties of this genus. All *Paradiplozoon* specimens collected from Europe are included in Clade 1 alongside all species of *Diplozoon*. However, there are also specimens collected from Asia and Africa in this clade. The close affiliation between European *Paradiplozoon* species and *Diplozoon* taxa has been shown to mirror the close relationship of their hosts [4, 29, 48, 49], further supporting the idea that all of these species belong to a single genus (Genus 1 (*Diplozoon*) as discussed before). However, from a geographical origin, members of this group are widely spread and the river systems in which they occur do not share a common ocean or region.

Considering the *Paradiplozoon* species in Clade 2 (Fig. 1), we can see that most species are from Asian (China and Turkey) origin, except for *Paradiplozoon krugerense* Dos Santos & Avenant-Oldewage, 2016 from Africa and the two species for which no locality data are currently available (*Paradiplozoon barbi* (Reichenbach-Klinke, 1951) and *Paradiplozoon* sp.). For the latter two species, *P*. *barbi* has only been recoded from Malaysia and the sequence authors are based in Malaysia, thus it can be assumed that the specimens were collected from this country. Similarly, the authors of the unidentified *Paradiplozoon* sp. are based in China, making it highly probable that both these taxa are of Asian origin. When the Asian and African characters of the *Paradiplozoon* species in Clade 2 are considered, in conjunction with the possibility of two additional diplozoid genera within this clade as discussed before (Genus 2 (*Paradiplozoon*) and Genus 3), a possible integrated picture emerges. Genus 2 (*Paradiplozoon*) is then entirely of China-Malaysia origin, consisting of *P*. *barbi*, *P*. *yunnanense* and members of the ‘*P. hemiculteri*-complex’. This could mean that the ancestor to these species may have originated in the South China Sea or other parts of the Pacific Ocean. Genus 3 would then consist of *Paradiplozoon* species collected from Africa, Turkey and China. As both *P*. *krugerense* and *Paradiplozoon bingolense* Civáňová et al., 2013 were collected from river systems which eventually enter the Indian Ocean, this may indicate that members of this proposed genus have an Indo-Pacific ancestor, further supported by the close phylogenetic relation to the members of Genus 2. Collection of *A*. *polycotyleus* also took place from a river system entering the Indian Ocean, supporting the possible Indo-Pacific origin of all species in Clade 2. An appropriate designation for Genus 3 may then be *Indodiplozoon* based on the possibility of an ancestor from the Indo-Pacific (Additional file 2: Table S3).

## Species complexes

### *Paradiplozoon homoion*-complex

From the results presented here (Additional file 2: Table S2, Fig. 1), three species of diplozoids in Clade 1 are very closely related when considering the ITS2 rDNA fragment. These are *P*. *homoion*, *P. gracile*, and *Paradiplozoon skrjabini* (Akhmerov, 1974). *Paradiplozoon homoion* was originally described from *Rutilus rutilus* (Linnaeus, 1758), but has since been collected from more than 30 species of cyprinids, displaying uncharacteristically loose host specificity [55]. The identity of *P*. *gracile* has changed several times since it was originally described as *Diplozoon gracile* Reichenbach-Klinke, 1961, being reclassified as *Diplozoon homoion gracile* Oliver & Reichenbach-Klinke, 1973 (supported by Prost [56]), then moved to the genus *Paradiplozoon* as *Paradiplozoon homoion gracile* (Reichenbach-Klinke, 1961), and currently known as *P*. *gracile*. As can be seen from the shift of this species from a distinct taxon, to a subspecies, back to a standalone species, the validity of this species has been the topic of some debate. Most of the aforementioned taxonomic changes were based on morphological differences and similarities observed between *P*. *gracile* and *P*. *homoion*. Using allozyme analysis, Le Brun et al. [57] suggested that these species could be differentiated, while both Sicard et al. [29] and Matejusová et al. [4] concluded *P*. *gracile* a synonym for *P*. *homoion* based on identical ITS2 rDNA even when collected from a range of hosts. Matejusová et al. [55] attempted to revise the relation of these species, referring to them as the ‘*P*. *homoion*-complex’ and concluded that *P*. *gracile* should be considered a *species inquirenda* as they were unable to detect a robust criterion for distinguishing the two supposed taxa based on detailed sclerite morphology and ITS2 sequences. As such, most studies in which *P*. *gracile* was studied using molecular tools did not publish separate sequence data as they were identical to those obtained for *P*. *homoion*. However, two sequences have been made public through GenBank by Wang & Yue (unpublished) for the *cox*1 and ITS2 markers of *P. gracile*. For the ITS2 rDNA fragment assessed above, *P*. *homoion* and *P*. *gracile* sequences are identical, while 0.76% separate representative sequences of these species when *cox*1 mtDNA is considered. This supports the findings of other studies indicating no resolution between members of this species complex using ITS2 rDNA, but may showcase the ability of *cox*1 mtDNA in differentiating intrinsically close taxa. This may be supported by the idea that the allozyme difference between *P*. *homoion* and *P*. *gracile* indicate local partial genetic differentiation, but that there is either still enough gene flow between them to maintain rDNA similarity, or that their separation is due to a recent barrier and thus sequence variation is delayed [48].

Looking at the produced topology and the calculated distances using ITS2 rDNA fragments (Additional file 2: Table S2, Fig. 1), *P. skrjabini* collected in Russia, Japan, and China [58] are also closely related to the widely distributed *P. homoion*. Similar to *P*. *homoion* and *P*. *gracile*, *P*. *skrjabini* has also been collected from several host species (7 to date), and thus shows relaxed host specificity. According to Shimazu et al. [58], *P*. *skrjabini* is morphologically very similar to *P*. *homoion*, only differing by the absence of anterior joining sclerites in the latter species. These three species form a well-supported clade, with most of the sequences for *P*. *skrjabini* sister to those of *P*. *homoion*, *P*. *gracile* and a single sequence for *P*. *skrjabini*, the two groupings separated by 1.02–1.32%. The sequence for *P*. *skrjabini* (KP340974) which groups with *P*. *homoion* and *P*. *gracile* is yet unpublished, but is noted from the same host genus as sequence LC050528 (*P*. *skrjabini* from *Leuciscus waleckii* (Dybowski, 1869)). Also, KP340974 is only separated from sequences for *P*. *homoion* and *P*. *gracile* by 0.3%, while it is separated from other *P*. *skrjabini* sequences by 1.17–1.32%. From these results, two possibilities arise. First, that *P*. *skrjabini* also forms part of the ‘*P*. *homoion*-complex’, bringing the total number of species in the complex to three, with intraspecific distances of up to 1.32% based on ITS2 rDNA. This is supported by the 1.5% robust limit designated earlier to discriminate between species based on ITS2 rDNA. The second possibility would be that the identification of KP340974 was incorrect and truly represented either *P*. *homoion* or *P*. *gracile*. This would mean that the ‘*P*. *homoion*-complex’ consists only of *P*. *homoion* and *P*. *gracile*, with *P*. *skrjabini* a very closely related sister taxon, possibly indicating a subspecies. The latter scenario is supported by Khotenovsky [3], who lists *Leuciscus baicalensis* (Dybowski, 1874) (from which KP340974 (*P. skrjabini*) were collected) as a host of *P*. *homoion*. However, when considering hosts, both *P*. *homoion* and *P*. *skrjabini* parasitize members of the genus *Leuciscus*, while all three diplozoid species have been recorded from *Phoxinus* hosts. Additionally, Sicard et al. [48] analysed an unidentified diplozoid parasite collected from *Tribolodon hakonensis* (Günther, 1877) in Japan, the same host and locality from which diplozoids were collected and identified as *P*. *skrjabini* by Shimazu et al. [58]. Sicard et al. [48] recorded a close molecular relationship of these parasites from *T*. *hakonensis* to *P*. *homoion* collected in Europe, similar to what is seen in the generated topology (Fig. 1), suggesting that they were in fact studying *P*. *skrjabini*. Unfortunately, this sequence data were not published and are unavailable for analyses.

It is thus most likely that all three the species in question form part of the ‘*P*. *homoion*-complex’ coined by Matejusová et al. [55]. The wide geographical distribution of this cryptic ‘*P*. *homoion*-complex’ may even reach into Africa as Sicard et al. [48] noted that sequences obtained for diplozoid parasites collected from *Labeo coubie* Rüppell, 1832 in Ivory Coast were genetically “strictly similar” (*sic*.) to those of *P*. *homoion* collected in Europe. Unfortunately, this sequence data were also not published, and as such the data cannot be analysed. It is thus necessary to study representatives of all the diplozoids which could be attributed to these three species in order to further elucidate their taxonomic status and that of the entire species complex. In this regard, the use of additional markers, like *cox*1 mtDNA (which appears to enable discrimination between *P*. *homoion* and *P*. *gracile* unlike ITS2 rDNA), would be informative. Newly implemented morphometric techniques, such as the study of diplozoid sclerites using scanning electron microscopy (SEM) [59, 60], may also be useful in this regard.

### *Paradiplozoon hemiculteri*-complex

From the results presented here (Additional file 2: Table S2, Fig. 1), six species of diplozoids in Clade 2 are very closely related when considering ITS2 rDNA, forming a purely Chinese *Paradiplozoon* clade. These are *P*. *hemiculteri*, *P*. *opsariichthydis*, *P*. *parabramisi*, *Paradiplozoon diplophyllorchidis* (Jiang et al., 1985), *Paradiplozoon parapeleci* (Jiang et al., 1984) and *Paradiplozoon jiangxiense* (Jiang et al., 1985). All these diplozoids were collected in adjacent river basins, the Yangtze and Pearl River Basins, but from a number of different host species. Sequences for all these species form a well-supported clade, with a grouping of select sequences for *P*. *hemiculteri* sister to a grouping of the remaining *P*. *hemiculteri* sequences and sequences of the other five species. The first grouping contains only *P*. *hemiculteri* sequences produced from a single study [32] and all from the same host, *Hemiculter leucisculus* (Basilewsky, 1855), the type-host of the species, with intraspecific distances of 0–2.34%. Sequences in the second grouping (containing all six species in question), were produced during several independent studies, some of which are unpublished, and from seven hosts including *H*. *leucisculus*, with intraspecific distances of 0–0.66%. Between these two groupings, 1.71–3.05% uncorrected p-distances were calculated, with the highest values between sequences both identified as *P*. *hemiculteri* (DQ098892 and KY124652) and collected from the same host, indicating that there is something amiss regarding the sequence data in this clade. Jirsová et al. [32], the authors for the sequences of the first grouping containing only *P*. *hemiculteri*, noted that the divergence between the sequences in their study were negligible, with only 0.1–0.3% separating them. But the data produced here (Additional file 2: Table S2), which were generated using data retrieved from GenBank, contradict this with values up to 2.34%, which is much higher than the 1.5% robust limit designated earlier to discriminate between species based on ITS2 rDNA. However, if this robust limit of 1.5% is used to consider the two groupings, the second grouping within the Chinese *Paradiplozoon* clade should represent a distinct taxon, as it is separated from the first grouping by 1.71–3.05%.

As such, two possibilities arise. First, that all six *Paradiplozoon* species in this clade represent members of a ‘*P*. *hemiculteri*-complex’ which has a very large sequence divergence of up to 3.05% for ITS2 rDNA. This does not seem likely, as this would call into question the validity of all diplozoid species that are separated by less than 3.05% ITS2 rDNA sequence divergence. Secondly, that some form of error has occurred in the data capturing of the *Paradiplozoon* sequences from China. Jirsová et al. [32] noted that their collections took place at the type-locality of *P*. *hemiculteri*. Thus, it is fair to expect that their sequences do indeed represent *P*. *hemiculteri* from *H*. *leucisculus*. That implies that the identification of both *P*. *hemiculteri* and their hosts collected by Gao et al. [40] and Xi, Buga & Li (unpublished) may be incorrect [32]. In this case, there may be two species complexes in China, a ‘*P*. *hemiculteri*-complex’ in the Pearl River Basin (based on the high divergence of the sequences of Jirsová et al. [32]), and a ‘*Paradiplozoon parabramisi*-complex’ in the Yangtze River Basin (*P*. *opsariichthydis*, *P*. *parabramisi*, *P*. *diplophyllorchidis*, *P*. *parapeleci*, *P*. *jiangxiense* and *P*. *hemiculteri* sequences by Gao et al. [40] and Xi, Buga & Li (unpublished)). Jirsová et al. [32] noted that morphological descriptions of Chinese diplozoids presented a hinderance to their study, with the inaccurate identification and lack of morphological records in genetic studies compounding the issue. Again, the use of additional markers like *cox*1 mtDNA alongside expanded morphometric analyses [59, 60] may be informative to resolve this question.

## Species identification hurdles

Recently, three diplozoid species were genetically characterised from India, producing the first sequence data for diplozoid parasites from the subcontinent [35, 36]. Two of these species, *D*. *guptai* and *Paradiplozoon kashmirense* (Kaw, 1950) have been described and recorded from the subcontinent and its native fish. But the third species, *Paradiplozoon aegyptense* (Fischthal & Kuntz, 1963) was described and previously recorded only from Africa and native African fish. This species was collected from indigenous hosts in India and was distinct from all other diplozoid species by at least 5.62% (ITS2 rDNA). Thus, the diplozoids collected by Ahmad et al. [35, 36] from *Carassius carassius* (Linnaeus, 1758) and *Schizopyge niger* (Heckel, 1838) represent a diplozoid taxon not previously sequenced and little doubt exists about its genetic distinctness. However, due to the outdated literature used by the authors to identify the diplozoid taxa in their study, still referring to the species as *Diplozoon aegyptensis*, in addition to *P*. *aegyptense* only recorded from native African cyprinids in the past, the identification of these diplozoids as *P*. *aegyptense* is unconvincing. This is further complicated by the findings of Sicard et al. [48] as mentioned above, where diplozoids collected from Ivory Coast were noted to be genetically “strictly similar” (*sic*.) to *P*. *homoion*. Sicard et al. [48] collected these specimens from *L*. *coubie*, a recorded host of *P*. *aegyptense*, unlike those of Ahmad et al. [35, 36]. As such, several possibilities arise. First, that *P*. *aegyptense* is a synonym for *P*. *homoion* based on the findings of Sicard et al. [48], and as such the identification by Ahmad et al. [35, 36] is incorrect as the obtained sequence is not similar to those for *P*. *homoion*. Secondly, that some form of human error occurred during the study of Sicard et al. [48] with sequences for *P*. *homoion* mistakenly attributed to diplozoids from the Ivory Coast, allowing for the possibility that the identification by Ahmad et al. [35, 36] was correct. Finally, it is also possible that *P*. *homoion* can infect native African fish hosts and that the diplozoids collected from *L*. *coubie* by Paperna [7, 8] were in fact *P*. *homoion*. This would mean that the diplozoids studied by Sicard et al. [48] do not truly represented *P*. *aegyptense*. Without genetic characterisation of diplozoids collected from the type-host in the type-locality to confirm the status of *P*. *aegyptense*, this mystery persists. Additionally, *P*. *kashmirense* has been noted as a synonym of *E*. *nipponicum* [61] (possibly *E*. *kamegaii* [22]). Given the distinct nature of the sequences currently available for *P*. *kashmirense* and their large distance from those for *E*. *nipponicum*, either the identification or the synonymizing with *E*. *nipponicum* was incorrect. This suggests that much work is still needed to fully incorporate diplozoid taxa from the subcontinent in taxonomic and systematic studies.

Another example of problematic species identification can be seen with the diplozoid specimens studied by Zhang et al. [28]. *Eudiplozoon* specimens were collected from *C*. *auratus* in China and may thus represent *E*. *nipponicum* which was recently confirmed to infect *Carassius* sp. in Japan [22]. Zhang et al. [28] produced ITS2 rDNA sequence data for these worms and note that they differed by 5–6% from *E*. *kamegaii*. Unfortunately, they did not publish these sequences and thus cannot be directly compared to the recently published sequence data for *E*. *nipponicum*. Fortunately, *cox*1 mtDNA could be mined from the mitogenomes published by Zhang et al. [28] and a comparison with both *E*. *nipponicum* and *E*. *kamegaii* was possible. *Cox*1 data for the unidentified species of *Eudiplozoon* were distinct from other *cox*1 sequences for the same genus (9.36–15.78%). As discussed, the *cox*1 data for *E*. *nipponicum* and *E*. *kamegaii* do not provide clear taxonomic results, with either two or four taxa possible. Nevertheless, the large distance of the data produced by Zhang et al. [28] from other *Eudiplozoon cox*1 data, alongside the large ITS2 distance from *E*. *kamegaii* (which is almost double the distance between *E*. *kamegaii* and *E*. *nipponicum*), suggest that the specimens do not represent *E*. *nipponicum*, but instead a cryptic, possibly undescribed species of *Eudiplozoon* as suggested by Zhang et al. [28]. Interestingly, Sicard et al. [48] also collected a diplozoid from *C*. *auratus* (the same host as Zhang et al. [28]) in Japan (the same locality as Nishihira & Urabe [22]) and noted it to be 2.74% (ITS2 rDNA) from *E*. *kamegaii*. The ITS2 distance, locality, and host of the samples by Sicard et al. [48] relate more closely to *E*. *nipponicum* [22] than the unidentified *Eudiplozoon* from Zhang et al. [28]. But again, the sequence data of Sicard et al. [48] were not published and cannot be critically assessed. The apparent dissimilarly between *Eudiplozoon* specimens from *C*. *auratus* in Japan and China is puzzling but may provide a good opportunity to study the morphometric and genetic variation between these host-parasite groups. This may also help resolve the apparent uncertainty around the identity of *Carassius* species in these areas as Nishihira & Urabe [22] note the hosts of *E*. *nipponicum* they collected as *C*. *buergeri grandoculis* and *Carassius* sp. (common names nigorobuna and ginbuna, respectively), which are synonyms for *C*. *auratus* and *Carassius langsdorfii* Temminck & Schlegel, 1846 respectively according to FishBase [62].

Similarly, Zhang et al. [28] collected an unidentified *Sindiplozoon* sp. from several host species in China, differing by 3% from available ITS2 rDNA sequence data for this genus. The taxonomy of this genus is very understudied, with between two [3, 39] and six species [63] considered valid. The distance between published *Sindiplozoon* ITS2 sequence data and that produced by Zhang et al. [28], support that the latter does not represent *Sindiplozoon ctenopharyngodoni* (Ling, 1973) and instead a diplozoid not previously sequenced (most likely *S*. *diplodiscus* as discussed earlier). Like the sequence data for the unidentified *Eudiplozoon* sp., the ITS2 rDNA data for the unidentified *Sindiplozoon* sp. were not published and micrographs for taxonomically important structures of these samples included in the publication are not sufficient to accurately describe or identify the species. However, the fact that the ITS2 rDNA sequence data produced by Zhang et al. [28] for *P*. *opsariichthydis* were identical to previously published data for this species, confirms the credibility of their data and suggests the potential for at least one new species (*Eudiplozoon*) and the genetic characterisation of at least one cryptic species (*Sindiplozoon*). All the aforementioned circumstances highlight the importance of generating comprehensive, accurate, and integrated data that include morphological and genetic aspects for known taxa, as well as new and cryptic species, preferably from the type-hosts and localities to prevent unnecessary confusion. Additionally, the defining of a more accurate and robust system for the successful identification and differentiation of diplozoid species is sorely needed and should be actively pursued.

## Inconsistencies and ethics

During the collection of data and material for this review, several inconsistencies and unconvincing notions were uncovered. Apart from those mentioned in the previous sections, there are some which relate more to the procedural and ethical aspects of molecular taxonomy which should also be addressed. The first is the use of outdated generic placement when referring to diplozoid species. To date, the most complete and widely accepted revision of the Diplozoidae was by Khotenovsky [3]. This work is difficult to source and was published in totality in Russian, with information for species occurring in only the Amur and Palaearctic regions translated and published in English by Pugachev et al. [36]. Thus, it is understandable that some authors are not aware of the revised taxonomy of some species. However, when Khotenovsky [3] is cited as part of the literature used in a study, and the new designation of the species is ignored without reason, it creates unnecessary confusion and uncertainty.

Another point of concern is the publication and release of sequence data. As mentioned, several sequences are currently available on GenBank even though they do not refer to any published work. This has both negative and positive aspects. On the one hand, such sequence data are available and can be included in the work of other researches to obtain larger data sets and often more comprehensive results. But having access to data which have not and possibly may never be peer-reviewed and published, often raises more questions than answers. The present authors acknowledge that this is an easy error as they are responsible for GenBank sequence HF565162 which was also not linked to a published work until now. An attempt to reconcile and complete missing or incomplete data was attempted here and, as per the previous sections, were explained and captured (Additional file 1: Table S1). However, some information for the more cryptic data could not be deduced, and one can only hope that the authors of those sequences will at some point publish their findings and make the information available.

Finally, it would appear that some studies have been published in duplicate in different journals, with identical sequence data designated different accession numbers. This creates unnecessary confusion as it is unclear whether these sequences are from separate individuals and can be used to infer intraspecific variation or stability, or are simply duplicates of the same data.

## Final suggestions and plea for voucher material

As is clear from the points discussed and the molecular data reviewed, we have only scratched the surface of the molecular study of the Diplozoidae. However, by summarising, reviewing and scrutinising the available information, it can now be attempted to steer this endeavour in the right direction. The first point to consider is the information obtained from online data repositories. As has been illustrated here, the data obtained from such sources are not always complete or related to a published, peer-reviewed work. It is also clear that using some of the data may create more confusion than it resolves. As such, we propose that the merit and value of data be assessed before being included in analyses. In doing this, it should be possible to limit the confusion these sequences may cause. For example, the sequence for an unidentified *Paradiplozoon* sp. by Kadirden (Arken) and Cheng (Yue) (MN892630-MN892639) clearly represent a distinct diplozoid taxon and thus contain valuable information, but they have not been published and thus very little information can be gathered for them. This means that any conclusions drawn from them in the present manuscript are entirely speculative and cannot be considered anything else.

Another consideration is the proper study and cataloguing of morphological data. Looking at several of the papers mentioned here, morphological study of the specimens in question are glossed over. Whether this is because the authors are not familiar with diplozoid taxonomy, whether the morphology is neglected (as with many purely molecular studies), or because the addition of descriptive morphology would make the study too large, none of these scenarios are conducive to the holistic study of any organism. This is because the outcome is that sequence data often remain unidentified, some species are incorrectly identified, or that sequence data or supporting information are never properly published and lost. These outcomes force future studies to make speculative conclusions (as was done here) with little hope of reaching a satisfactory conclusion. They also make the revisiting of specimens and whole studies necessary to re-assess the morphology of the specimens in question, which is often impractical if not impossible, also ending with speculative and unsatisfactory conclusions. Data produced and discussed in dissertations, theses, and conference proceedings, but never actively peer-reviewed, published, or submitted to data repositories, are also usually not obtainable and do not allow re-examination. The only way to prevent these issues is to either accurately record the full taxonomic details of the specimens in the publication accompanying sequence data, or to provide voucher material which can be re-examined at a later stage, bringing us to the final point of discussion.

A prominent issue which has been made clear in this review is the lack of accurate historical data and the completeness thereof. Recent descriptions of diplozoid taxa have included not only the full morphological description of the taxa, but also representative sequence data for ITS2 rDNA. This means that the sequence data for these species were likely obtained from the same cohort as the other taxonomic information used to describe the species and can thus be considered the holo- or paragenetype for the species [64]. Unfortunately, this is only the case for a handful of species [30, 31, 41, 50, 51], with all those produced before 2012 lacking genetic data for the type-material. Naturally, one could not expect the description of *D*. *paradoxum* to contain genetic data in 1832, but this illustrates the need for those producing species data now to consider the future. This means that studies involving the collection and study of organisms should make contingency arrangements, as far as realistically possible, for the future re-examination of material. To this end, we propose the following suggestions: (i) that voucher material be designated whenever diplozoid specimens are collected; (ii) that voucher material consists of as many formats as possible (e.g. 70% EtOH, 96% EtOH, GAP slides); (iii) that voucher material be housed in more than one location; (iv) that the housing institution/collection is freely accessible to researchers globally (longstanding and established museums); and (v) the location and accession codes of the voucher material be properly recorded in the relevant publication. Following these guidelines, it may be possible to overcome some of the hurdles exposed in the present review. For many of the studies mentioned, if vouchers were deposited in a museum collection and material could be re-examined with both morphological and molecular tools, their results may have been substantiated or revised, allowing for a more accurate taxonomic view of the Diplozoidae. These vouchers could then also be designated as genophore vouchers following Pleijel et al. [65], allowing for future genetic studies. Hologenophores (material for genetic study obtained from holotype specimen) and paragenophores (material for genetic study obtained from paratype specimens) [65] are not available for most diplozoids, even for species for which genetypes were included in their description, and thus if any other markers would need to be assessed for these species in the future, one would have to collect fresh material. These would then represent syngenophore (material for genetic study obtained from specimens of a specific species, but from a different occasion and/or locality) material, from which genetypes (neogenetype, topogenetypes, etc.) could be retrieved. The latter would be the only way to obtain genetic information of species described in the past as the submission of material fit for molecular analyses is not common for monogeneans. However, collecting new material allows for the designation of neotype material for species of which the original type material is missing or lost, like was done for *P*. *hemiculteri* [32] and *E*. *nipponicum* [22], but this is not always possible.

## Conclusions

In conclusion, it is clear that a substantial amount of morphological and genetic data are needed before an accurate study of the taxonomy and evolutionary history of diplozoid species can be achieved. But, by identifying the causes of confusion and uncertainty from currently available information, they can be avoided in future studies. An inclusive, multidisciplinary approach to such studies will need to be incorporated, alongside the proper collection and preservation of voucher material. This should include accurate morphological identification (using traditional as well as modern taxonomic techniques), the use of molecular techniques with various markers, and the proper deposition and curation of both morphological and genophore vouchers, all actively complementing each other. Additionally, the co-phylogenetic study of diplozoids and their hosts may add valuable insight into the taxonomy and evolution of both groups. Finally, by summarising the work that has been done and analysing the produced data in a wholistic manner, it is hoped that the present review will serve as a reference point for diplozoid study and promote integrative taxonomy in future endeavours.

## Supplementary information

**Additional file 1: Table S1.** Table containing all sequence data available for the family Diplozoidae, as compiled from public data repositories (GenBank) and literature.

**Additional file 2: Table S2.** Sequence divergence (average uncorrected p-distance in %) for diplozoid taxa based on ITS2 rDNA. **Table S3.** Proposed genus designation based on p-distance and phylogenetic results.

## Data Availability

Data used in this review were obtained from online data repositories and published literature. No new data were generated. Alignments and other datasets composed in this review will be provided on request.

## Abbreviations

BI: Bayesian inference
BLASTn: Nucleotide Basic Local Alignment Search Tool
*cox*1: cytochrome *c* oxidase subunit 1
EBI: European Bioinformatics Institute
GTR: general time reversible model
ITS1: first internal transcribed spacer region
ITS2: second internal transcribed spacer region
MCMC: Markov chain Monte Carlo generations
MAFFT: multiple alignment using fast Fourier transform
ML: maximum likelihood
mtDNA: mitochondrial DNA
P: parsimony
rDNA: ribosomal DNA
SEM: scanning electron microscopy
