# Supplementary material for: Review on the molecular study of the Diplozoidae: analyses of currently available genetic data, what it tells us, and where to go from here
Source: Parasit Vectors. 2020 Oct 30;13:539. doi: 10.1186/s13071-020-04417-3 (PMC7602351; doi:10.1186/s13071-020-04417-3)
Supplement: Supplementary file 1 — Additional file 1: Table S1. Table containing all sequence data available for the family Diplozoidae, as compiled from public data repositories (GenBank) and literature. [file 13071_2020_4417_MOESM1_ESM.pdf]

Additional File 1: Table S1. Table containing all sequence data available for the family Diplozoidae, as compiled from public data repositories (GenBank) and literature.

Diplozoid species	Host species[a]	Region	Accession number	Reference	Locality	Taxonomic synonyms used
Afrodiplozoon polycotyleus (Paperna, 1973)	*Labeobarbus marequensis* (Smit, 1841)	ITS2	**LT719088**	[1]	Lutanandwa River, Limpopo Province, South Africa	–
Diplozoon guptai Fayaz & Chishti, 2000	*Schizopyge niger* (Heckel, 1838)	ITS2	**AF973618**[b]	[2, 3]	Dal and Anchar Lake, Kashmir Valley, India	*Schizothorax niger* Heckel, 1838
Diplozoon paradoxum Nordmann, 1832	*Abramis brama* (Linnaeus, 1758)	28S	**AF131717**	[4]	Sweden	*Diplozoon* sp.[f]
		COI	**KP340976**	Wang & Yue[c]	China	–
		ITS2	**KP326299**	Wang & Yue[c]	China	–
			**AJ563372**	[5]	Kyjovka River, Czech Republic	
		ITS2 + 28S	**AF369759**	[6]	Scamandre pond, Camargue, France [7]	*Diplozoon paradoxum* Von Nordmann, 1832
Eudiplozoon kamegaii Nishihira & Urabe, 2020	*Cyprinus carpio* Linnaeus, 1758	18S	**AJ287510**	[8]	–	*Eudiplozoon nipponicum* (Goto, 1891)
			**MF579987**	[9][d]	Mert Lake, İğneada, Kırklareli, Turkey	*Eudiplozoon nipponicum* (Goto, 1891)
		28S	**AF311703**	[10]	France	*Eudiplozoon nipponicum* (Goto, 1891) *Diplozoon nipponicum* Goto, 1891
			**AF382037**	[11]	Czech Republic	*Eudiplozoon nipponicum* (Goto, 1891)
		Cathepsin B[e]	**MF346929**	[12]	South Bohemia, Czech Republic	*Eudiplozoon nipponicum* (Goto, 1891)
		Cathepsin L1[e]	**KP793605**	[13]	South Bohemia, Czech Republic	*Eudiplozoon nipponicum* (Goto, 1891)

		Cathepsin L3[c]	**KP793606**	[13]	South Bohemia, Czech Republic	*Eudiplozoon nipponicum* (Goto, 1891)
		COI	**AY009163**	[10]	France	*Eudiplozoon nipponicum* (Goto, 1891) *Diplozoon nipponicum* Goto, 1891
			**LC517174–5**	[14]	Shiga, Japan	–
		Cysteine peptidase inhibitor (stefin)	**KY192529**	[15]	Mušov lowland reservoir, Czech Republic	*Eudiplozoon nipponicum* (Goto, 1891)
		ITS2	**AJ300710**	[16]	Morava River, Czech Republic	*Eudiplozoon nipponicum* (Goto, 1891)
			**DQ098895**	[17]	Tangxun Lake, China	*Eudiplozoon nipponicum* (Goto, 1891)
			**DQ098896**	[17]	Hong Lake, China	*Eudiplozoon nipponicum* (Goto, 1891)
			**DQ098897**	[17]	Danjiangkou Reservoir, China	*Eudiplozoon nipponicum* (Goto, 1891)
			**KP340975**	Wang & Yue[c]	China	*Eudiplozoon nipponicum* (Goto, 1891)
			**LC517162–72**	[14]	Shiga, Japan	–
		ITS2 + 28S	**AF369758**	[6]	Camargue, France [7]	*Eudiplozoon nipponicum* (Goto, 1891)
		Kunitz protease inhibitor (serine)	**MF346930**	[18]	South Bohemia, Czech Republic	*Eudiplozoon nipponicum* (Goto, 1891)
		Serine protease inhibitor (serpin)	**MF288891**	[19]	Mušov lowland reservoir, Czech Republic	*Eudiplozoon nipponicum* (Goto, 1891)
Eudiplozoon nipponicum (Goto, 1891)	*Carassius auratus* (Linnaeus, 1758)	COI	**LC517173, LC517176–7**	[14]	Shiga, Japan	*Carassius buergeri grandoculis* Temminck & Schlegel (nigorobuna)

		ITS2	**LC496176–82, LC496187**	[14]	Harie, Shin-asahi Town, Takashima City, Shiga, Japan	*Carassius buergeri grandoculis* Temminck & Schlegel (nigorobuna)
			**LC496183–6, LC496188–90**	[14]	Paddy field, Ohta, Shin-asahi Town, Takashima City, Shiga, Japan	*Carassius buergeri grandoculis* Temminck & Schlegel (nigorobuna)
	Carassius langsdorfii Temminck & Schlegel, 1846	ITS2	**LC496174–5**	[14]	Paddy field, Ohta, Shin-asahi Town, Takashima City, Shiga, Japan	*Carassius* sp. (ginbuna)
Eudiplozoon sp.	*Carassius auratus* (Linnaeus, 1758)	Mitogenome	**MG458328**	[20]	Tangxun Lake, Hubei Province, China	*Carassius auratus* complex Linnaeus, 1758
Inustiatus inustiatus (Nagibina, 1965)	*Hypophthalmichthys molitrix* (Valenciennes, 1844)	ITS2	**DQ098893**	[17]	Tangxun Lake, China	–
	Hypophthalmichthys nobilis (Richardson, 1845)	ITS2	**DQ098894**	[17]	Tangxun Lake, China	*Inustiatus aristichthysi* (Ling, 1973); *Aristichthys nobilis* (Richardson, 1845)
Paradiplozoon aegyptense (Fischthal & Kuntz, 1963)	*Schizopyge niger* (Heckel, 1838); *Carassius carassius* (Linnaeus, 1758)	ITS2 + 28S	**AF973617**[b]	[2, 3]	Dal Lake, Kashmir Valley, India	*Diplozoon aegyptensis* Fischthal & Kuntz, 1963; *Schizothorax niger* Heckel, 1838
Paradiplozoon barbi (Reichenbach-Klinke, 1951)	–	ITS2	**MN688771**	Ibrahim & Yahaya[c]	Malaysia	–
Paradiplozoon bingolense Civáňová et al., 2013	*Garra rufa* (Heckel, 1843)	ITS2	**HE653910**	[21]	Göynük Stream, Ilicalar-Bingöl, Turkey	*Paradiplozoon bingolensis* Civáňová et al., 2013
Paradiplozoon bliccae (Reichenbach-Klinke, 1961)	*Squalius fellowesii* (Günther,1868)	ITS2	**LT560257**	[22]	Doğanbaba Creek, Burdur, Turkey	–
	Blicca bjoerkna (Linnaeus, 1758)	ITS2	**AJ300712**	[16]	Morava River, Czech Republic	–

		ITS2 + 28S	**AF369761**	[6]	Scamandre pond, France [7]	*Diplozoon bliccae* Reichenbach-Klinke, 1961
Paradiplozoon diplophyllorchidis (Jiang et al., 1985)	*Zacco platypus* (Temminck & Schlegel, 1846)	ITS2	**DQ098891**	[17]	Danjiangkou Reservoir, China	*Paradiplozoon opsariichthydis* (Jiang et al., 1984)
Paradiplozoon gracile (Reichenbach-Klinke, 1961)	*Gobio acutipinnatus* Meńshikov, 1939	COI	**KP399596**	Wang & Yue[c]	China	–
		ITS2	**KP340973**	Wang & Yue[c]	China	–
Paradiplozoon hemiculteri (Ling, 1973)	*Hemiculter leucisculus* (Basilewsky, 1855)	18S	**KY640614**	Semi, Xi & Li[c]	Taihu Lake, Wuxi, China	–
		28S	**MN545903**	Zhang & Fan[c]	China	–
		ITS2	**DQ098884**	[17]	Bao'an Lake, China	–
			**DQ098887**	[17]	Tangxun Lake, China	–
			**DQ098888**	[17]	Danjiangkou Reservoir, China	–
			**DQ098892**	[17]	Hong Lake, China	–
			**KY124645–54**	[23]	Shaoguan, Guangdong Province, China	–
		18S + ITS1 + 5.8S + ITS2	**KY290757–61**	Xi, Buga & Li[c]	Wuxi, China	–
Paradiplozoon homoion (Bychowsky & Nagibina, 1959)	*Rutilus rutilus* (Linnaeus, 1758)	COI	**KP399595**	Wang & Yue[c]	China	–
		ITS2	**KP340972**	Wang & Yue[c]	China	–
			**AJ300715**	[16]	Morava River, Czech Republic	–
		ITS2 + 28S	**AF369760**	[6]	Scamandre pond, Camargue, France [7]	*Diplozoon homoion* Bychowsky & Nagibina, 1959
	Rhodeus amarus (Bloch, 1782)	ITS2	**MT028131**	Aydogdu[c]	Susurluk stream, Balikesir, Turkey	–
	Phoxinus phoxinus (Linnaeus, 1758)	ITS2	**AJ300715**	[16]	Morava River, Czech Republic	–

	Scardinius erythrophthalmus (Linnaeus, 1758)	ITS2 + 28S	**AF369760**	[6]	Scamandre pond, Camargue, France [7]	*Diplozoon homoion* Bychowsky & Nagibina, 1959
Paradiplozoon ichthyoxanthon Avenant-Oldewage 2014 in Avenant-Oldewage et al. 2014	*Labeobarbus aeneus* (Burchell, 1822)	COI	**HF565162**	[24]	Vaal River, South Africa	*Paradiplozoon sp.* [f]
		ITS2	**HF566124**	[24]	Vaal Dam and Vaal River, South Africa	–
Paradiplozoon jiangxiense (Jiang et al., 1985)	*Chanodichthys erythropterus* (Basilewsky, 1855)	ITS2	**DQ098885**	[17]	Tangxun Lake, China	*Paradiplozoon jiangxiensis* (Jiang et al., 1985)
						Cultrichthys erythropterus (Basilewsky, 1855)
Paradiplozoon kashmirense (Kaw, 1950)	*Cyprinus carpio* Linnaeus, 1758; *Schizopyge niger* (Heckel, 1838); *Schizothorax esocinus* Heckel, 1838; *Schizothorax curvifrons* Heckel, 1838; *Carassius carassius* (Linnaeus, 1758)	ITS2 + 28S	**AF973616**[b]	[2, 3]	Dal Lake, Kashmir Valley, India	*Diplozoon kashmirensis* Kaw, 1950; *Schizothorax niger* Heckel, 1838; *Cyprinus carpio cummunis* Anonymous
	–	ITS2 + 28S	**MF460994**	Sofi & Ahmad [c]	Kashmir Valley, India	*Diplozoon kashmirensis* Kaw, 1950
Paradiplozoon krugerense Dos Santos & Avenant-Oldewage, 2016	*Labeo rosae* Steindachner, 1894	ITS2	**LT574865**	[25]	Flag Boshielo Dam, Limpopo, South Africa	–
Paradiplozoon megan (Bychowsky & Nagibina, 1959)	*Squalius cephalus* (Linnaeus, 1758)	ITS2	**AJ300711**	[16]	Morava River, Czech Republic	*Leuciscus cephalus* (Linnaeus, 1758)
Paradiplozoon nagibinae (Glaser, 1965)	*Ballerus ballerus* (Linnaeus, 1758)	ITS2	**AJ563371**	[5]	Kyjovka River, Czech Republic	*Abramis ballerus* (Linnaeus, 1758)
Paradiplozoon opsariichthydis (Jiang et al., 1984)	*Opsariichthys uncirostris* (Temminck & Schlegel, 1846)	ITS2	**DQ098890**	[17]	Danjiangkou Reservoir, China	–

			**MH794184–8**	Jirsova, Koubkova, Jirounkova, Vorel, Ding, Gelnar & Kasny[c]	Zhenjiang, Shaoguan, China	–
		Mitogenome	**MG458327**	[20]	Danjiangkou, Hubei Province, China	–
Paradiplozoon parabramisi (Ling, 1973)	*Parabramis pekinensis* (Basilewsky, 1855)	ITS2	**DQ098883**	[17]	Tangxun Lake, China	–
			**DQ098889**	[17]	Hong Lake, China	–
	Megalobrama amblycephala Yih, 1955	ITS2	**DQ098886**	[17]	Tangxun Lake, China	–
Paradiplozoon parapeleci (Jiang et al., 1984)	*Pseudolaubuca sinensis* Bleeker, 1864	ITS2	**DQ098882**	[17]	Danjiangkou Reservoir, China	*Parapelecus argenteus* Günther, 1889
Paradiplozoon pavlovskii (Bychowsky & Nagibina, 1959)	*Leuciscus aspius* (Linnaeus, 1758)	ITS2	**AJ300714**	[16]	Morava River, Czech Republic	*Aspius aspius* (Linnaeus, 1758)
Paradiplozoon sapae (Reichenbach-Klinke, 1961)	*Ballerus sapa* (Pallas, 1814)	ITS2	**AJ300713**	[16]	Morava River, Czech Republic	*Abramis sapa* (Pallas, 1814)
Paradiplozoon skrjabini (Akhmerov, 1974)	*Leuciscus baicalensis* (Dybowski, 1874)	ITS2	**KP340974**	Wang & Yue[c]	China	
	Leuciscus waleckii (Dybowski, 1869)	ITS2	**LC050528**	[26]	Bolshaya Ussurka River, Primorsky Region, Russia	–
	Phoxinus steindachneri Sauvage, 1883	ITS2	**LC050524**	[26]	Metoba River, Matsumoto City, Nagano, Japan	–
	Rhynchocypris lagowskii (Dybowski, 1869)	ITS2	**LC050529**	[26]	Narva River, Primorsky Region, Russia	–
	Rhynchocypris oxycephalus (Sauvage & Dabry de Thiersant, 1874)	ITS2	**LC050525**	[26]	Takami River, Kotsugawa, Nara, Japan	*Phoxinus oxycephalus* (Sauvage & Dabry de Thiersant, 1874)
	Tribolodon hakonensis (Günther, 1877)	ITS2	**LC050521**	[26]	Ueda City, Nagano, Japan	–

			**LC050522**	[26]	Sai River, Azumino City, Nagano, Japan	–
			**LC050523**	[26]	Tenryu River, Ina City, Nagano, Japan	–
			**LC050527**	[26]	Lake Abashiri, Abashiri City, Hokkaido, Japan	–
	Tribolodon sachalinensis (Nikolskii, 1889)	ITS2	**LC050526**	[26]	Lake Abashiri, Abashiri City, Hokkaido, Japan	–
Paradiplozoon vaalense Dos Santos et al. 2015	*Labeo umbratus* (Smith, 1841)	ITS2	**HG423142**	[27]	Vaal Dam and Vaal River, South Africa	–
Paradiplozoon yunnanense Fan et al., 2018	*Sikukia gudgeri* (Smith, 1934)	ITS2	**MF775370**	[28]	Jinghong Basin, Lancang-Mekong River, Yunnan, China	*Paradiplozoon yunnanensis* Fan et al., 2018
	Sikukia stejnegeri Smith, 1931	ITS2	**MT336740**	Zhang[c]	China	*Paradiplozoon yunnanensis*
	–	ITS2	**KT781100**	[29][d]	China	*Paradiplozoon yunnanensis*
Paradiplozoon sp.	*Barilius barila* (Hamilton, 1822); *Barilius bendelisis* (Hamilton, 1807)	28S	**KU519493**	Chetry[c]	Arunachal Pradesh, India	–
Paradiplozoon sp.	–	ITS2	**MN892630, MN892637–8**	Kadirden & Cheng[c]	China[f]	–
Paradiplozoon sp.	–	ITS2	**MN892631–6, MN892639**	Arken & Yue[c]	China[f]	–
Sindiplozoon ctenopharyngodoni (Ling, 1973)	*Ctenopharyngodon idella* (Valenciennes, 1844)	ITS2	**DQ098898**	[17]	Tangxun Lake, China	–
Sindiplozoon sp.	*Spinibarbus hollandi* Oshima, 1919; *Parabramis pekinensis* (Basilewsky, 1855); *Mylopharyngodon piceus* (Richardson, 1846)	Mitogenome	**MG458326**	[20]	Mangtang stream, Hunan Province, China	–

[a] Host names as given by FishBase [30].

[b] Sequences from Ahmad et al. 2015 not available from online databases, only recorded in text.

[c] Sequences are published to online databases but are not published in literature. Authors are given as cited online.

[d] Publication only as a conference abstract.

[e] Cysteine peptidases.

[f] Designated following conclusions of the present study.

References

1. Přikrylová I, Mašová Š, Gelnar M, Matla MM, Tavakol S, Luus-Powell WJ. Redescription of the genus *Afrodiplozoon* Khotenovski, 1981 and its only known species *Afrodiplozoon polycotyleus* (Paperna, 1973) (Monogenea: Diplozoidae) using a combined multidisciplinary approach. Parasitol Int. 2018;67:245–52.

2. Ahmad F, Fazili KM, Sofi TA, Waza AA, Rashid R. Comparative molecular characterization of three *Diplozoon* species from fishes of Kashmir Valley. Agric Adv. 2015;4:65–83.

3. Ahmad F, Fazili KM, Sofi TA, Sheikh BA, Waza AA, Rashid R, et al. Morphological and molecular characterization of *Diplozoon kashmirensis*; *D. aegyptensis* and *D. guptai* collected from fishes of Kashmir Valley- India. Fish Aquac J. 2015;06:1000147.

4. Mollaret I, Jamieson BGM, Justine J-L. Phylogeny of the Monopisthocotylea and Polyopisthocotylea (Platyhelminthes) inferred from 28S rDNA sequences. Int J Parasitol. 2000;30:171–85.

5. Matejusová I, Koubková B, Cunningham CO. Identification of European diplozoids (Monogenea, Diplozoinae) by restriction digestion of the ribosomal RNA internal transcribed spacer. J Parasitol. 2004;90:817–22.

6. Sicard M, Desmarais E, Lambert A. Caractérisation moléculaire des populations de Diplozoidae sur cinq espèces de Cyprinidae: nouvelles données sur la spécificité parasitaire. Comptes Rendus l’Académie des Sci Paris, Sci la Vie. 2001;324:709–17.

7. Sicard M, Desmarais E, Vigneux F, Shimazu T, Lambert A. Molecular phylogeny of the Diplozoidae (Monogenea, Polyopisthocotylea) parasitizing 12 species of Cyprinidea (Teleostei): new data about speciation. In: Combes C, Jourdane J, editors. Taxonomy, ecology and

evolution of metazoan parasites. Perpignan: PUP; 2003. p. 199–211.
8. Littlewood D, Olson PD. Small subunit rDNA and the Platyhelminthes: Signal, noise, conflict and compromise. In: Littlewood D, Bray RA, editors. Interrelationships of the Platyhelminthes. 1st edition. London: CRC Press; 2001. p. 262–78.
9. Kırcalar F, Turgay E, Yardımcı RE, Soylu E, Akmirza A. İğneada mert gölü sazan balıklarında (*Cyprinus carpio*) *Eudiplozoon nipponicum* (Gotto, 1891)'un moleküler identifikasyonu. In: Ulusal Su Ürünleri Sempozyumu. Sinop; 2017. p. 206.
10. Jovelin R, Justine JL. Phylogenetic relationships within the polyopisthocotylean monogeneans (Platyhelminthes) inferred from partial 28S rDNA sequences. Int J Parasitol. 2001;31:393–401.
11. Olson PD, Littlewood DTJ. Phylogenetics of the Monogenea - evidence from a medley of molecules. Int J Parasitol. 2002;32:233–44.
12. Jedličková L, Dvořáková H, Dvořák J, Kašný M, Ulrychová L, Vorel J, et al. Cysteine peptidases of *Eudiplozoon nipponicum*: a broad repertoire of structurally assorted cathepsins L in contrast to the scarcity of cathepsins B in an invasive species of haematophagous monogenean of common carp. Parasit Vectors. 2018;11:142.
13. Jedličková L, Dvořáková H, Kašný M, Ilgová J, Potěšil D, Zdráhal Z, et al. Major acid endopeptidases of the blood-feeding monogenean *Eudiplozoon nipponicum* (Heteronchoinea: Diplozoidae). Parasitology. 2016;143:494–506.
14. Nishihira T, Urabe M. Morphological and molecular studies of *Eudiplozoon nipponicum* (Goto, 1891) and *Eudiplozoon kamegaii* sp. n. (Monogenea; Diplozoidae). Folia Parasitol (Praha). 2020;67:018.
15. Ilgová J, Jedličková L, Dvořáková H, Benovics M, Mikeš L, Janda L, et al. A novel type I cystatin of parasite origin with atypical legumain-binding domain. Sci Rep. 2017;7:17526.
16. Matejusová I, Koubková B, D'Amelia S, Cunningham CO. Genetic characterization of six species of diplozoids (Monogenea; Diplozoidae). Parasitology. 2001;123:465–74.
17. Gao Q, Chen MX, Yao WJ, Gao Y, Song Y, Wang GT, et al. Phylogeny of diplozoids in five genera of the subfamily Diplozoinae Palombi, 1949 as inferred from ITS-2 rDNA sequences. Parasitology. 2007;134:695–703.
18. Jedličková L, Dvořák J, Hrachovinová I, Ulrychová L, Kašný M, Mikeš L. A novel Kunitz protein with proposed dual function from

Eudiplozoon nipponicum (Monogenea) impairs haemostasis and action of complement in vitro. Int J Parasitol. 2019;49:337–46.
19. Roudnický P, Vorel J, Ilgová J, Benovics M, Norek A, Jedličková L, et al. Identification and partial characterization of a novel serpin from *Eudiplozoon nipponicum* (Monogenea, Polyopisthocotylea). Parasite. 2018;25:61.
20. Zhang D, Zou H, Wu SG, Li M, Jakovlić I, Zhang J, et al. Three new Diplozoidae mitogenomes expose unusual compositional biases within the Monogenea class: implications for phylogenetic studies. BMC Evol Biol. 2018;18:133.
21. Civáňová K, Koyun M, Koubková B. The molecular and morphometrical description of a new diplozoid species from the gills of the *Garra rufa* (Heckel, 1843) (Cyprinidae) from Turkey—including a commentary on taxonomic division of Diplozoidae. Parasitol Res. 2013;112:3053–62.
22. Unal MC, Innal D, Civáňová K, Stavrescu-Bedivan MM, Koubková B, Ozmen O, et al. Identification of two gill parasites *Lamproglena compacta* (Copepoda: Lernaeidae) and *Paradiplozoon bliccae* (Monogenea: Diplozoidae) from endemic Aegean chub (*Squalius fellowesii*). Bull Eur Assoc Fish Pathol. 2017;37:135–47.
23. Jirsová D, Ding X, Civáňová K, Jirounková E, Ilgová J, Koubková B, et al. Redescription of *Paradiplozoon hemiculteri* (Monogenea, Diplozoidae) from the type host *Hemiculter leucisculus*, with neotype designation. Parasite. 2018;25:4.
24. Avenant-Oldewage A, le Roux LE, Mashego SN, van Vuuren BJ. *Paradiplozoon ichthyoxanthon* n. sp. (Monogenea: Diplozoidae) from *Labeobarbus aeneus* (Cyprinidae) in the Vaal River, South Africa. J Helminthol. 2014;88:166–72.
25. Dos Santos QM, Avenant-Oldewage A. The description of a new diplozoid species, *Paradiplozoon krugerense* n. sp., from *Labeo rosae* Steindachner, 1894 and *Labeo congoro* Peters, 1852 in the Kruger National Park, South Africa with notes on the effect of water quality on its. Hydrobiologia. 2016;777:225–41.
26. Shimazu T, Kobayashi K, Tojo K, Besprozvannykh VV, Ogawa K. *Paradiplozoon skrjabini* (Monogenea, Diplozoidae), an Ectoparasite on the Gills of Freshwater Fishes (Cyprinidae, Leuciscinae) of Japan and Primorsky Region, Russia: a Morphological and Molecular Study. Bull Natl Museum Nat Sci Ser A. 2015;41:137–54.
27. Dos Santos QM, van Vuuren BJ, Avenant-Oldewage A. *Paradiplozoon vaalense* n. sp. (Monogenea: Diplozoidae) from the gills of moggel,

Labeo umbratus (Smith, 1841), in the Vaal River System, South Africa. J Helminthol. 2015;89:58–67.
28. Fan L-X, Meng F-Y, Bai J-P, Xu W-J, Wang X. *Paradiplozoon yunnanensis* n. sp. (Monogenea, Diplozoidae) from *Sikukia gudgeri* (Cyprinidae, Barbinae) in southwest China. Parasite. 2018;25:46.
29. Fan L, Bai J, Meng F, Wang J, Xu W, Wang X. Identification of a new species of genus *Paradiplozoon* Achmerov, 1974 from the gills of *Sikukia gudgeri* (Smith, 1931) (Cyprinidae) from China. In: 8th International Symposium on Monogenea. 2017. p. 59.
30. Froese R, Pauly D. FishBase. www.fishbase.org. Accessed 6 Sep 2020.
